# Effects of Irrigation with Treated Wastewater on Root and Fruit Mineral Elements of Chemlali Olive Cultivar

**DOI:** 10.1155/2014/973638

**Published:** 2014-06-11

**Authors:** Saida Bedbabis, Béchir Ben Rouina, Makki Boukhris, Giuseppe Ferrara

**Affiliations:** ^1^Laboratory of Environment and Biology of Arid Area, Department of Life Science, Faculty of Sciences, P.O. Box. 802, 3018 Sfax, Tunisia; ^2^Laboratory of Improvement of Olive and Fruit Trees' Productivity, Olive Tree Institute, P.O. Box. 1087, 3000 Sfax, Tunisia; ^3^Dipartimento di Scienze del Suolo, della Pianta e degli Alimenti, University of Bari “Aldo Moro,” Via Amendola 165/A, 70126 Bari, Italy

## Abstract

Twenty-year-old “Chemlali” olive trees trained to vase and rainfed were investigated in either “on” (2004) or “off” (2003) year. A randomized block design with three blocks and three treatments was used and each experimental plot consisted of nine olive trees. Three treatments were applied: (1) rainfed conditions (RF, used as control treatment); (2) irrigation with well water (WW); and (3) irrigation with treated wastewater (TWW). Irrigation with TWW led to a significant increase of root N, P, Ca, Zn, Mn, Na, and Cl concentrations, in particular in the on-year. Data showed significant differences, between the two years, for the concentration of the mineral elements in the roots, with general lower values in the on-year, probably as a consequence of nutrients movement upward in the tree. Fruit N, P, K, Zn, Mn, and Cl contents were significantly higher in TWW irrigated trees with respect to both RF and WW trees, whereas similar values for Ca, Mg, Na, and Cl contents were measured for WW and TWW irrigated trees. The irrigation with TWW allowed to reuse problematic waters and to save nutrients inputs in the olive orchard thus moving towards a more sustainable management of olive orchards in countries where water is the major limiting factor for agriculture.

## 1. Introduction

In arid and semiarid regions, water resources of good quality are becoming more and more scarce and are being allocated with priority for urban water supply. Taking into account the scarcity of conventional water resources, due to water demand increases both for human consumption and for agricultural use, the reuse of saline, brackish, and treated wastewater (TWW) could be a realistic way for reducing water shortage, as it has been demonstrated in many countries in the Mediterranean basin. The TWW can constitute a reliable water and nutrients source for crops [1] and its use for irrigation reduces the amount of nutrient-rich waters returned to rivers or sea; but its use can have controversial impacts, especially because of the potential heavy metals risk for plant growth, agriculture products [2], and physical-chemical properties of the soils [3]. The reuse of TWW in Tunisia can either satisfy the increasing water requirements of agriculture or constitute a tool to preserve freshwater resources for human consumption. Currently, the effluent used for irrigation is mainly obtained after biological treatments (secondary treatment). However, this effluent differs from freshwater for salinity, pH, and concentrations of microelements and nutrients; all parameters are generally higher in TWW than in freshwater [4].

Tunisia is in the northeastern part of Africa, which is considered one of the driest regions in the world [5]. Agriculture is the major mainstay of the Tunisian economy, and the cultivation of olive trees constitutes one of the principal sectors of agriculture in terms of economic weight. In fact, about 65 million olive trees are spread over 1.6 million hectares [6]. Chemlali is the main olive cultivar grown in northern and central Tunisia and accounts for 80% of Tunisia's oil production [7].

Some studies have focused on the effects of irrigation with treated wastewater and the application of olive mill wastewater on mineral elements content in olive leaves [8, 9], but very limited studies have investigated the effect of TWW or saline water on mineral elements content in roots and fruits [10, 11]. This aspect could be very important for either yield or nutrients inputs to the olive orchard. The aim of this work was to study the effect of a two-year irrigation period with TWW on mineral elements in both olive roots and fruits in trees in on- and off-year yield.

## 2. Material and Methods

### 2.1. Field Conditions, Climatic Data, Plant Material, and Irrigation Management

**Figure 1:**
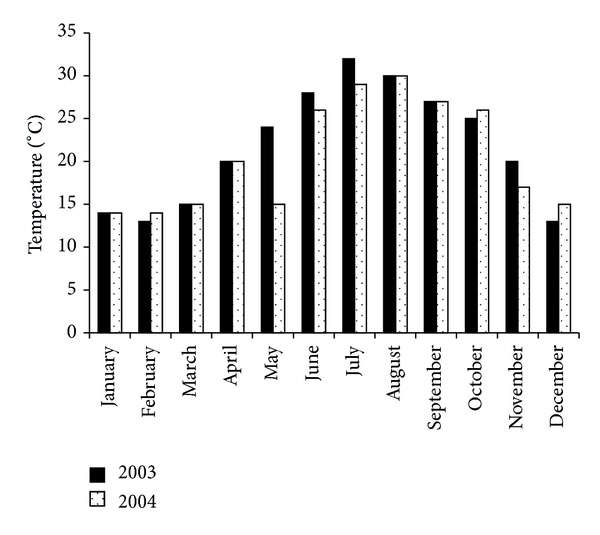
Monthly mean temperatures (°C) registered at the experimental site in 2003 and 2004.

**Figure 2:**
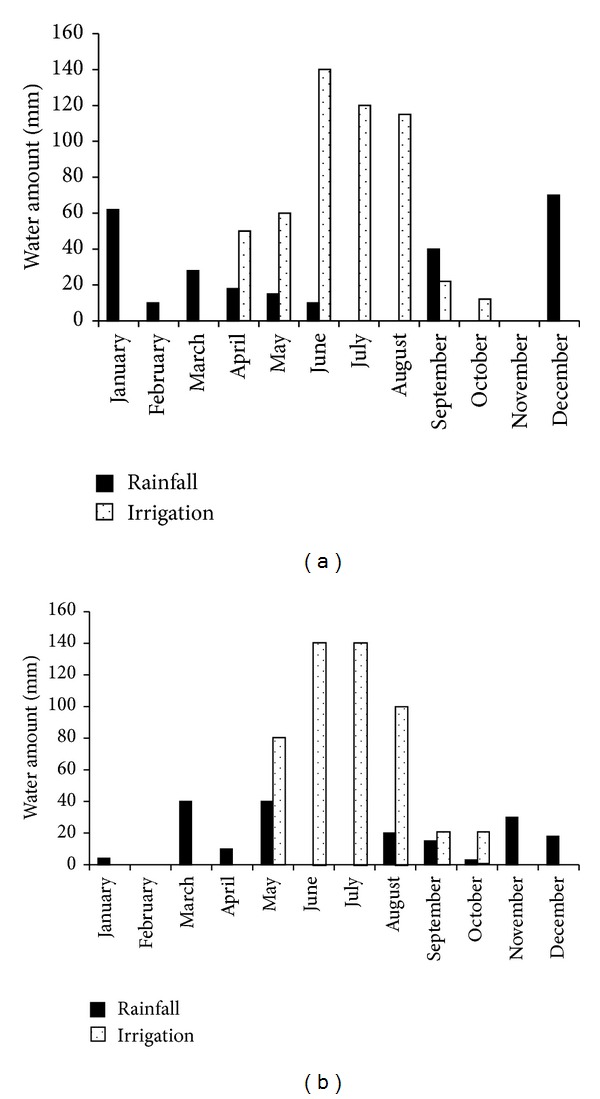
Monthly rainfall and irrigation values (mm) registered at the experimental site in 2003 (a) and 2004 (b).

The experiment was carried out at El Hajeb experimental station, located in the region of Sfax, (34°43 N, 10°41 E) in Central-Eastern Tunisia. The climate is Mediterranean with a mean annual precipitation, which occurs mostly in autumn and winter, of 276 mm and a mean air temperature of 32°C. Mean temperature values and rainfall and irrigation amount (mm) are reported in Figures 1 and 2, respectively. The study was carried out in 2003 (“off” year) and 2004 (“on” year) in an olive orchard planted in 1987 with “Chemlali” olive trees. The soil is sandy according to USDA soil texture classification.

Chemlali is an olive cultivar with alternate bearing habit, and twenty-year-old trees (24.0 × 24.0 m spaced), in “on” (heavily fruiting trees in 2004) and “off” (slightly fruiting trees in 2003) year, trained to vase and rainfed were selected. A randomized block design with three blocks and three treatments was used and each experimental plot consisted of nine olive trees bordered by a double guard row. Three treatments were applied: (1) rainfed conditions (RF, used as control treatment); (2) irrigation with well water (WW); and (3) irrigation with treated wastewater (TWW). The WW was obtained from a well near the experimental station, whereas the TWW from a biological treatment process. The waters were analyzed once a year for physical and chemical parameters.

Drip irrigation was made with four drip nozzles (two per side) set in a line along the rows, at 0.5 m from the trunk. Trees were daily irrigated with approximately 4 L h^−1^, with a seasonal irrigation volume of 150 mm. During the experimental years, irrigation was performed from April to May and from October to December.

### 2.2. Root Sampling and Mineral Elements Analysis

Fine roots were sampled in the four seasons of each year (winter, spring, summer, and autumn), put in paper bags and stored in a portable cooler for the determination of nutrients concentration. Successively, the roots were dried in an oven (60°C) and analyzed according to the methods described by Pauwels et al. [12]. Mineral elements (N, P, K, Na, Cl, Ca, Mg, Zn, and Mn) analysis was carried out after dry-ashing at 450°C in a muffle oven (HEROTEC) and digestion of the ashes with 1 M of HNO_3_. Total nitrogen was determined with the Kjeldahl method. K and Na were determined by atomic emission spectrophotometry (JENWAY PFP7, Milan, Italy). Ca, Mg, Zn, and Mn were analyzed by atomic absorption spectrophotometry (PerkinElmer A Analyst 300, PerkinElmer Inc., Willesley, MA, USA). P in roots was determined by a vanadomolybdate colorimetric procedure with a JENWAY 6405 UV/Vis Spectrophotometer (Milan, Italy). Finally, Cl was determined titrimetrically with AgNO_3_ [13].

### 2.3. Fruit Sampling and Mineral Elements Analysis

Healthy fruits were hand-harvested in 2004 during ripening (summer) and at technological maturity (autumn). Fruits were sampled in triplicate from all the olive trees of each treatment (each sample 1 kg). Pulp and stone were separated, put in paper bags, and dried at 60°C for 72 h. Successively, both tissues were ground and mineral analyses were carried following the same methods previously described for roots.

### 2.4. Yield and Oil Content Determination

In the middle of December olives were hand-harvested to guarantee the accuracy and weighed to obtain the yield. Healthy fruit samples (2 kg) were harvested in triplicate from all the olive trees of each block. Samples were immediately carried to the laboratory for oil extraction. The oil content was determined by Soxhlet extraction and was expressed as a percentage of dry olive paste weight.

### 2.5. Statistical Analysis

All collected data were subjected to the analysis of variance, with the three treatments as the independent variables. Statistical analyses were carried out with the SPSS 10 for Windows (SPSS, Inc., Chicago, IL, USA). The mean values of all parameters were compared using the LSD test at 0.05, 0.01 and 0.001 *P* levels.

## 3. Results and Discussions 

### 3.1. Chemical Characteristics of Waters

The results of the chemical analysis of both TWW and WW used in the experiment are presented in Table 1. The pH of the TWW and WW was 7.60 and 7.95, respectively, thus falling within the limits for crop irrigation, which range from 6.0 to 9.0 [14].

The electrical conductivity (EC) was 6.30 dS m^−1^ for TWW and 4.70 dS m^−1^ for WW, indicating, respectively, a high and moderate level of salinity [15, 16]. Cl concentration was higher than the threshold values, as reported by Chartzoulakis [17] in the guidelines for olive irrigation. Generally, TWW contained higher amounts of N, P, and K with respect to WW, and these elements are considered essential for plant growth and development. Both chemical and biological oxygen demands (COD and BOD) were below the Tunisian thresholds for water reuse (90 and 30 mg L^−1^, resp.).

### 3.2. Root Mineral Elements

#### 3.2.1. Nitrogen

Nitrogen (N) concentration in the roots varied between 0.75 and 1.90% of dry matter in WW and RF samples, whereas a significant increase of N was reported in roots of trees irrigated with TWW (Table 2). This significant increase was the consequence of N supply by TWW and its higher absorption by fine roots. A significant decrease of N was reported during summer both in on- and off-year in all the treatments (Table 2) and a significant increase of this element was reported in leaves (from 1.32% to 1.52%, data not showed). This decrease can be attributed to N migration from roots to leaves and successively to the fruits. In general, results showed significant differences for all the periods of the year between on- and off-year trees. A significant roots accumulation of N was reported at spring in the off-year and at autumn in the on-year. The high concentration can be explained by high roots N absorption during spring (off-year) and its translocation/absorption during autumn (on-year). In both years, the accumulation of N was higher in TWW irrigated trees with respect to RF and WW ones.

These data suggest, both for off- and on-year, the N needs of the trees for the vegetative activity (off-year) and the fruit growth (on-year). The high N concentration also detected at autumn in the on-year seems to indicate a possible N downward movement after the ripening, possibly for the needs, in the subsequent year, of the vegetative activity. As a practical consequence, in the on-year N application can be limited to spring and early summer until pit hardening [18], and possibly also after harvest for an optimal vegetative activity in the successive year [11]. In the off-year, a major winter-spring application of N should be required in order to allow a good vegetative growth [11].

#### 3.2.2. Phosphorus

The mean P concentration ranged from 0.04 to 0.13%, 0.04 to 0.07%, and 0.03 to 0.06% for TWW, WW, and RF roots, respectively (Table 2). Results showed that irrigation with TWW led to a significant increase of roots P concentration. The significant increase was attributed to high root P absorption as a consequence of P accumulation in soil solution. In a recent study in greenhouse conditions, the root P concentration of “Improved Nabali” and “Manzanillo” significantly increased after wastewater application [10].

In the off-year, a significant decrease of root P concentration was reported at summer (Table 2), similarly to what reported by Bustan et al. [11]. This decrease can be explained by P migration from roots to other plant parts for the vegetative growth (role of P in cell division), as shown by the significant increase of shoots length in off-year trees [19].

A significant peak of P concentration was reported in winter of the on-year (2004). This peak was attributed to (i) high root activity in autumn for P absorption and (ii) P accumulation by TWW supply. The high P concentration reported in winter during the on-year can suggest P application for an optimal yield because the role of P in the reproductive processes is well known and has been recently confirmed by various works [20, 21].

In on-year, slight P concentrations were reported at summer and autumn. This may be due to the high demand of P from the fruit (sink) for oil biosynthesis [19] and consequently high P concentrations were measured in fruits (from 0.09 to 0.28% d.m).

#### 3.2.3. Potassium

In off- and on-year, trees irrigated with TWW presented a significant decrease of roots K concentrations compared to WW treatment. The decrease was probably the consequence of (i) larger amounts of Na and Cl supplied by TWW compared to WW and (ii) an increase in root Na content, since Na replaces the nutrients and competes with K for binding and absorption sites of the fine roots. Our results are in agreement with previous findings in different olive cultivars [10, 22]. The observed reduction in K concentration at the current investigation, which resulted in a low K/Na ratio, may suggest a mechanism by which olive trees achieve an ionic balance following uptake of Na in roots [10]. Lower Na concentrations compared to K concentrations were reported in olive leaves in the TWW treatment [19]. The decrease of roots K can be also explained by high Ca supply by TWW that enhanced the selectivity for the uptake and the transport of K for the other plant organs (leaves, stems) with respect of Na.

The significant decrease of root K concentrations at summer and autumn may be due to different plant organs uptake for vegetative and reproductive growth and high demand for either vegetative activity or fruit development and oil biosynthesis. In fact, an increase of K concentration has been reported both in leaves [19] and in fruits. With these data, K supply in autumn and winter in on- and off-year can stimulate the reproductive and vegetative growth, respectively.

#### 3.2.4. Calcium

Roots Ca ranged from 1.23 to 3.32% of roots in TWW irrigated trees (Table 3). Ca concentrations were significantly higher in TWW and WW irrigated trees with respect to RF ones. The roots Ca accumulation in irrigated roots can be due to (i) high Ca input by TWW and (ii) roots Ca absorption for the ability of olive tree to limit the salts absorption. High root Ca concentration was associated with a general lower root Na concentration. It may be due to Ca competition with Na for the binding sites. Results are in contrast with data of Al Absi et al. [10] in different olive cultivars and this can be explained by TWW composition.

In the off-year, a significant decrease of root Ca concentration was reported at autumn, probably because Ca transport from roots to the other plant organs for late vegetative growth. These data are in agreement with the significant increase of shoot length measured in autumn for off-year olive trees [19]. In the on-year, Ca concentration was significantly low in the winter period because of the high demand of Ca for fruit development and oil biosynthesis.

#### 3.2.5. Magnesium

Root Mg concentrations were significantly higher in TWW and WW trees with respect to RF ones (Table 3). The difference can be explained by Mg input through irrigation. Results also revealed that water salinity partially affected the root Mg concentration with similar values both in TWW and WW trees. Our results are in agreement with previous findings in three olive cultivars: Nabali, Improved Nabali, and Manzanillo [10].

In the off-year, a significant reduction of root Mg concentration was detected in summer. This result can be explained by Mg transport from roots to the leaves for photosynthesis. High net photosynthesis values were reported in off-year olive trees (14 *μ*mol/m^2^/s). On the contrary, in the on-year lower Mg values were measured in spring.

#### 3.2.6. Sodium and Chloride

The contents of sodium (Na) and chloride (Cl) are reported in Table 3. A significant accumulation of both elements was reported in TWW trees as compared to WW and RF ones. The increase of both elements was the consequence of the input by TWW irrigation. Our results are in agreement with previous findings in Nabali and Improved Nabali cultivars [10]. The root concentrations of both elements increased as the salinity of water increased suggesting that root Na and Cl absorption increased with increasing concentration of these salts in the water and in the root zone (500 mg kg^−1^ and 283 mg kg^−1^ for Na and Cl, resp., in the upper layers of irrigated TWW soil). The high concentration of Na in soil solution of trees irrigated with TWW has been correlated to Na content in leaves and roots [10].

The results of Cl and Na accumulation in “Chemlali” roots are in agreement with previous works in olive [22, 23] describing ions exclusion and retention of Cl, as well as Na, in roots. Salt tolerance of Chemlali cultivar can be based on (i) root salts absorption and (ii) the ability to limit the salts transport from the roots to the shoots. Salt tolerance is mainly associated with salt-exclusion mechanisms operating in the roots, preventing salt translocation rather than salt absorption [24–26] by holding Na and Cl at the root level and limiting the accumulation of these ions into actively growing shoots. Olive trees are less sensitive to Cl uptake and transport to the shoot with respect to Na [23]. Ca is also supposed to play an important role in Na exclusion and retention mechanisms, which may be an important ability for survival in saline conditions [27, 28].

#### 3.2.7. Zinc and Manganese

Zn and Mn contents are reported in Table 4. Root Mn and Zn concentrations of “Chemlali” significantly increased after irrigation with TWW compared to WW. Our results are in agreement with previous findings in Nabali cultivar [10]. Root Zn and Mn values increased as a consequence of the high concentration of these heavy metals in the TWW and in the root zone [10].

### 3.3. Fruit Mineral Elements Content

Mineral elements contents of olive fruits in the on-year are reported in Table 5. N, P, and K contents are significantly higher in TWW irrigated trees than in WW and RF trees. The increase may be due to the nutrients input by TWW that induced high available N, P, and K in soil solution and consequent higher plant uptake. A trend of a lower K content in fruits of RF trees with respect to saline water irrigation has been previously observed for Picual cultivar [29].

Nutrients accumulation may be due to N and K migration from leaves to fruits for amino acids and proteins synthesis. Connor and Fereres [30] found a large amount of K in olive fruits, similarly to what was detected in our work, where an increase of K was measured from fruit growth up to maturity (Table 5). Nitrogen (N) is essential for the construction of primary metabolites such as amino acids, proteins, and nucleotides as well as numerous secondary metabolites [11]. The significant decrease of fruits N and P contents reported at harvest (autumn) may be the consequence of the increased size of the drupe.

Fruits from RF trees showed lower Ca and Mg content than WW and TWW fruits thus indicating that irrigation positively affected these nutrients content. TWW irrigation did not cause changes of Ca and Mg content with respect to WW.

Cl and Na contents in WW and TWW irrigated fruits were significantly higher with respect to RF trees. It was probably because uptake and mobility of Cl and Na are closely linked to uptake fluxes and water movement inside the tree [31]. Both Na and Cl contents were generally higher at harvest (autumn) with respect to the ripening period (summer).

Finally, significant fruit Zn and Mn accumulations were reported in TWW irrigated trees as compared to WW or RF trees. The higher Zn and Mn content reported at harvest may be the consequence of the transport from leaves to fruits for the ripening process.

### 3.4. Yield and Oil Content

Olive cultivars produce large amounts of pollen grains [32], and significant differences are noticeable between an on- and an off-year [33], with higher values in the former. This difference is then evident in the consequent yield/tree; in fact, in our trial, in the off-year (2003), olives production was 86.4 kg tree^−1^ in TWW treatment compared to 27.6 kg tree^−1^ in WW treatment (*P* ≤ 0.01). In the on-year (2004), olives production was again significantly higher for TWW with 154 kg tree^−1^ compared with 90.3 kg tree^−1^ of WW. Although it is reported in other studies that saline waters might reduce yield [16, 22, 34] compared to control conditions (good quality water), in the present two-year study we did not observe such a negative effect. The higher yield obtained in TWW irrigated trees was probably a consequence of the presence of nutrient elements such as N, P, and K, and the irrigation treatment worked as fertigation. In a recent 9-year study [29] on irrigation of olive trees with different waters (0.5, 5, and 10 dS m^−1^), no differences were observed in annual and cumulated yield among treatments after irrigation with saline waters.

Oil content does not constitute a criterion of oil quality determination but especially a criterion to be considered during the varietal selection [35]. The average oil content of the olives harvested from the TWW irrigated trees decreased from 51.71 to 44.82% (d.w.) from the on- to the off-year.

## 4. Conclusions

TWW irrigation increased N, P, Ca, Zn, and Mn and decreased the K contents in roots with respect to WW irrigation. The root salts concentration increased as the salinity of water increased suggesting that root Na and Cl absorption increased with increasing concentration of these elements in the irrigation water and in the root zone. Cl and Na accumulation in “Chemlali” roots may be due to ions exclusion and retention of Cl as well as Na in roots. Fruit N, P, K, Zn, and Mn contents were significantly higher in TWW irrigated trees with respect to RF and WW trees. With regards of trees in off- and on-year, significant differences have been observed for almost all the elements either in roots or fruits. The data collected do indicate the elements movement in the trees for sustaining either the vegetative or reproductive growth of the tree, depending whether in an on- or off-year. The use of TWW for irrigation can either allow to reuse waters not drinkable or to save nutrients inputs in the olive orchard (less fertilizers). In the on-year, TWW irrigated trees presented a higher concentration of nutrients and this could be important for the successive vegetative season (off-year). In conclusion, our results can give useful indications for a more rational nutrition schedule of olive trees saving both mineral and water inputs towards a more sustainable management of the olive orchard.

## Figures and Tables

**Table 1:** Chemical properties of the well water (WW) and treated wastewater (TWW) used for irrigation in the experimental olive orchard.

Characteristics	WW	TWW	Tunisian limits
pH	7.95 ± 0.10	7.60 ± 0.11	6.50–8.50
EC (dS m^−1^)	4.70 ± 0.02	6.30 ± 0.03	7.00
TDS (g L^−1^)	1.51 ± 0.02	1.82 ± 0.01	2.00
HCO_3_^−^ (mg L^−1^)	288.50 ± 0.3	370.00 ± 0.20	600.00
SO_4_^2−^ (mg L^−1^)	87.50 ± 0.8	363.00 ± 1.50	1000
N total (mg L^−1^)	—	58.80 ± 1.20	30.00
N–NO_3_^−^ (mg L^−1^)	1.11 ± 0.01	15.90 ± 0.05	
N–NH_4_^+^ (mg L^−1^)	2.24 ± 0.01	37.90 ± 0.01	
N–NO_2_^−^ (mg L^−1^)	0.08 ± 0.02	5.00 ± 0.01	
P total (mg L^−1^)	0.80 ± 0.11	10.30 ± 0.01	0.05
K^+^ (mg L^−1^)	30.00 ± 0.09	38.00 ± 0.02	50.00
Na^+^ (mg L^−1^)	355.00 ± 0.01	470.00 ± 0.02	300.00
Cl^−^ (mg L^−1^)	1580 ± 0.04	1999.00 ± 0.04	600.00
Ca^2+^ (mg L^−1^)	184.50 ± 0.01	95.80 ± 0.03	
Mg^2+^ (mg L^−1^)	126.20 ± 0.01	83.80 ± 0.02	
Pb^2+^ (mg L^−1^)	0	<0.004	0.10
Cd^2+^ (mg L^−1^)	0	<0.004	0.005
Zn^2+^ (mg L^−1^)	0.10 ± 0.01	0.42 ± 0.01	5.00
Mn^2+^ (mg L^−1^)	0.19 ± 0.01	0.50 ± 0.01	
SM (mg L^−1^)	4.30 ± 0.02	13.40 ± 0.03	
COD (mg L^−1^)	0	73.00 ± 0.11	90.00
BOD (mg L^−1^)	0	22.00 ± 0.04	30.00

Data represents mean values ± standard deviation.

**Table 2:** Roots N, P, and K contents in the three treatments: rainfed (RF), well water (WW), and treated wastewater (TWW) in “on” and “off” year.

Parameter	Year	Off (2003)			On (2004)			
		RF	WW	TWW	RF	WW	TWW	
N (% d.m.)	Winter	1.26^aC^	1.28^aC^	1.27^aC^	1.10^bC^	1.00^bC^	1.26^aC^	∗∗
	Spring	1.85^bA^	1.90^bA^	2.05^aA^	1.25^cB^	1.39^bB^	1.54^aB^	∗∗∗
	Summer	0.84^cD^	0.91^bD^	0.98^aD^	0.75^cD^	0.85^bD^	0.90^aD^	∗∗
	Autumn	1.35^cB^	1.40^bB^	1.58^aB^	1.40^cA^	1.50^bA^	1.65^aA^	∗∗

P (% d.m.)	Winter	0.05^bA^	0.07^bA^	0.11^aA^	0.06^bA^	0.07^bA^	0.13^aA^	∗∗
	Spring	0.05^aA^	0.07^aA^	0.06^aB^	0.04^bB^	0.06^aB^	0.07^aB^	∗∗
	Summer	0.03^aB^	0.04^aB^	0.04^aC^	0.05^bB^	0.06^bB^	0.08^aB^	∗∗
	Autumn	0.05^bA^	0.07^aA^	0.07^aB^	0.05^bB^	0.05^bB^	0.08^aB^	∗∗

K (% d.m.)	Winter	0.20^cD^	0.50^aB^	0.25^bC^	0.07^bC^	0.38^aD^	0.10^bC^	∗∗
	Spring	0.50^cA^	1.30^aA^	0.95^bA^	0.43^bA^	0.90^aA^	0.80^aA^	∗∗∗
	Summer	0.35^cB^	0.52^aB^	0.45^bB^	0.07^bC^	0.50^aB^	0.50^aB^	∗∗
	Autumn	0.28^cC^	0.40^bC^	0.47^aB^	0.15^cB^	0.45^bC^	0.55^aB^	∗∗

Small letters indicate significant differences (*P* ≤ 0.05) among treatments within each season and for each year according to LSD test. Capital letters indicate significant differences (*P* ≤ 0.05) among seasons within each treatment and for each year according to LSD test. Asterisk indicates significant differences between years (mean values of RF, WW, and TWW): significant at *P* ≤ 0.01 (∗∗) and *P* ≤ 0.001 (∗∗∗) according to LSD test.

**Table 3:** Roots Ca, Mg, Na and Cl contents in the three treatments: rainfed (RF), well water (WW), and treated wastewater (TWW) in “on” and “off” year.

Parameter	Year	Off (2003)			On (2004)			
		RF	WW	TWW	RF	WW	TWW	
Ca (% d.m.)	Winter	1.88^cA^	2.90^bC^	3.12^aA^	0.61^cD^	1.70^aD^	1.23^bD^	∗∗∗
	Spring	1.60^cB^	3.51^aA^	2.89^bB^	1.45^cB^	2.30^bB^	3.10^aB^	∗∗∗
	Summer	1.50^cC^	3.10^aB^	2.84^bB^	1.88^cA^	2.51^bA^	3.32^aA^	∗∗
	Autumn	1.09^bD^	1.58^aD^	1.58^aC^	0.97^cC^	2.40^aC^	1.50^bC^	∗∗

Mg (% d.m.)	Winter	0.40^bA^	0.45^bB^	0.55^aA^	0.27^bA^	0.48^aA^	0.43^aB^	∗∗
	Spring	0.28^cB^	0.51^aA^	0.45^bB^	0.18^bB^	0.42^aB^	0.40^aB^	∗∗
	Summer	0.16^cC^	0.46^aB^	0.33^bC^	0.32^cA^	0.52^bA^	0.70^aA^	∗∗∗
	Autumn	0.18^cC^	0.50^aA^	0.40^bB^	0.30^cA^	0.49^bA^	0.66^aA^	∗∗∗

Na (% d.m.)	Winter	0.005^cB^	0.02^bB^	0.21^aB^	0.005^cB^	0.09^bB^	0.31^aA^	ns
	Spring	0.009^cA^	0.12^bA^	0.19^aB^	0.007^cA^	0.13^bA^	0.23^aC^	ns
	Summer	0.004^cB^	0.03^bB^	0.29^aA^	0.009^cA^	0.05^bC^	0.27^aB^	ns
	Autumn	0.004^cB^	0.12^bA^	0.31^aA^	0.004^cB^	0.09^bB^	0.29^aB^	ns

Cl (% d.m.)	Winter	0.02^cA^	0.36^bA^	0.54^aA^	0.02^bA^	0.45^aA^	0.45^aB^	ns
	Spring	0.02^cA^	0.26^bB^	0.55^aA^	0.01^cA^	0.27^bB^	0.45^aB^	ns
	Summer	0.03^cA^	0.24^bB^	0.45^aB^	0.01^cA^	0.23^bB^	0.36^aC^	ns
	Autumn	0.02^cA^	0.27^bB^	0.54^aA^	0.01^cA^	0.27^bB^	0.54^aA^	ns

Small letters indicate significant differences (*P* ≤ 0.05) among treatments within each season and for each year according to LSD test. Capital letters indicate significant differences (*P* ≤ 0.05) among seasons within each treatment and for each year according to LSD test. Asterisk indicates significant differences between years (mean values of RF, WW and TWW): nonsignificant (ns) and significant at *P* ≤ 0.01 (∗∗) and *P* ≤ 0.001 (∗∗∗) according to LSD test.

**Table 4:** Roots Zn and Mn contents in the three treatments: rainfed (RF), well water (WW), and treated wastewater (TWW) in “on” and “off” year.

Parameter	Year	Off (2003)			On (2004)			
		RF	WW	TWW	RF	WW	TWW	
Zn (mg kg^−1^)	Winter	18^cA^	22^bA^	28^aA^	10^cA^	16^bA^	20^aA^	ns
	Spring	12^cB^	15^bB^	22^aA^	12^bA^	13^bA^	16^aA^	ns
	Summer	7^cC^	9^bC^	12^aB^	12^bA^	13^bA^	16^aA^	ns
	Autumn	11^cB^	22^bA^	25^aA^	13^bA^	14^bA^	17^aA^	∗∗

Mn (mg kg^−1^)	Winter	10^cB^	13^bB^	24^aB^	14^cA^	17^bA^	24^aA^	ns
	Spring	11^bB^	12^bB^	23^aB^	7^bB^	8^bB^	26^aA^	ns
	Summer	15^cA^	19^bA^	31^aA^	6^cB^	9^bB^	11^aC^	∗∗
	Autumn	18^cA^	22^bA^	32^aA^	5^cB^	9^bB^	18^aB^	∗∗∗

Small letters indicate significant differences (*P* ≤ 0.05) among treatments within each season and for each year according to LSD test. Capital letters indicate significant differences (*P* ≤ 0.05) among seasons within each treatment and for each year according to LSD test. Asterisk indicates significant differences between years (mean values of RF, WW and TWW): nonsignificant (ns) and significant at *P* ≤ 0.01 (∗∗) and *P* ≤ 0.001 (∗∗∗) according to LSD test.

**Table 5:** Fruits mineral elements content in the three treatments: rainfed (RF), well water (WW), and treated wastewater (TWW) in 2004 (“on” year).

Parameter	Period	RF	WW	TWW
N (% d.m.)	Summer	0.60^bA^	0.64^bA^	0.73^aA^
	Autumn	0.52^bB^	0.52^bB^	0.63^aB^

P (% d.m.)	Summer	0.17^cA^	0.22^bA^	0.28^aA^
	Autumn	0.09^cB^	0.12^bB^	0.17^aB^

K (% d.m.)	Summer	0.60^cB^	1.20^bB^	1.50^aB^
	Autumn	0.90^cA^	1.50^bA^	1.90^aA^

Ca (% d.m.)	Summer	1.73^bA^	3.00^aA^	2.80^aA^
	Autumn	0.95^bB^	1.75^aB^	1.50^aB^

Mg (% d.m.)	Summer	0.60^aA^	0.34^bA^	0.34^bA^
	Autumn	0.06^bB^	0.20^aB^	0.21^aB^

Na (% d.m.)	Summer	0.01^bA^	0.04^aB^	0.03^aA^
	Autumn	0.01^bA^	0.07^aA^	0.05^aA^

Cl (% d.m.)	Summer	0.04^bB^	0.20^aB^	0.22^aB^
	Autumn	0.08^bA^	0.32^aA^	0.27^aA^

Zn (mg kg^−1^)	Summer	4.50^cB^	6.72^bB^	9.77^aB^
	Autumn	10.00^cA^	18.65^bA^	25.40^aA^

Mn (mg kg^−1^)	Summer	4.00^cB^	5.70^bB^	7.82^aB^
	Autumn	6.00^cA^	8.46^bA^	10.00^aA^

Small letters indicate significant differences (*P* ≤ 0.05) among treatments within each season according to LSD test. Capital letters indicate significant differences (*P* ≤ 0.05) among seasons within each treatment according to LSD test.
